# Effect of Adding Guava (*Psidium guajava*) Epicarp Extract Flour on the Physicochemical, Textural, Colour and Sensory Properties of Frankfurters

**DOI:** 10.17113/ftb.59.02.21.7062

**Published:** 2021-06

**Authors:** Viviana Andrea Velasco-Arango, José Igor Hleap-Zapata, Luis Eduardo Ordóñez-Santos

**Affiliations:** Facultad de Ingeniería y Administración, Departamento de Ingeniería Universidad Nacional de Colombia – Sede Palmira, Carrera 32 No. 12-00, Palmira, Valle del Cauca, Colombia

**Keywords:** carotenoids, guava epicarp, nitrite reduction, frankfurters, fat oxidation

## Abstract

**Research background:**

The industrial transformation of tropical fruits, and in particular guava, generates a large quantity of by-products that are generally disposed of as organic waste. In these by-products a large quantity of bioactive substances is concentrated, such as carotenoids, which can be used for the partial substitution of nitrites in meat sausages without affecting their physicochemical, colour and conservation characteristics. Although there are some studies in this regard, there is still a lack of research, especially on guava residues, to verify this hypothesis. Therefore, the aim of this study is to investigate the influence of the components of the guava epicarp extract on the physicochemical, textural, colour and sensory properties of frankfurters.

**Experimental approach:**

There treatments were investigated (25, 50 and 75% nitrite replacement with guava epicarp flour extract), along with a control treatment without the extract. The physicochemical properties, colour coordinates, and texture parameters were analysed, and a sensory evaluation was also carried out. The treatment that gave the best results was subjected to stability analysis over 30 days.

**Results and conclusions:**

The addition of 25% guava epicarp flour extract gave the best results, since it did not affect the colouration of the sausages or their physicochemical and textural properties. Likewise, during the stability analysis over time, the parameters related to fat oxidation were not affected, and final products had a residual nitrite load of (23.6±0.2) mg/kg, lower than the maximum allowed (150 mg/kg).

**Novelty and scientific contribution:**

The partial substitution of the nitrites in frankfurters with the carotenoids from the guava epicarp flour added in a mass fraction up to 25% can be a viable alternative to produce sausages with lower content of nitrites, without affecting their colouration or shelf life. This is important for the meat industry, which favours the development of new products using substances of natural origin.

## INTRODUCTION

The production of tropical fruits has been growing worldwide in recent years. According to the data provided by FAO, the highest production of tropical fruits occurs in developing countries (*1*) such as Colombia with its geographical and biological diversity and varying climates that make up different ecosystems, which guarantee a high annual production of fruits and vegetables (*2*) including the significant production of guava (*Psidium guajava*). In 2019, according to data from the National Administrative Department of Statistics of Colombia (DANE) (*3*), 102 877 tonnes of guava were produced, destined for direct consumption as fresh fruit or for the development of multiple products. Agro-industrial transformation generates a large amount of by-products, basically epicarp and seeds, which are generally disposed of as organic waste. However, multiple studies confirm that guava epicarp is rich in bioactive compounds such as carotenoid pigments, antioxidants, phenols and vitamins (*4*, *5*).

Antioxidants play a fundamental role in human metabolism since they prevent oxidative cell alterations caused by free radicals, which minimises the possibility of contracting terminal diseases, such as cancer, Alzheimer’s or diabetes, along with other neurological and degenerative disorders (*6*, *7*). Fruits and vegetables are rich in bioactive compounds, including antioxidants (*8*), which makes them important for human diet. Therefore, in recent years, the trend has been to include them in the manufacture of numerous food products, including those of animal origin (*9*). Carotenoid pigments, fat soluble compounds that have red or orange colour, are among the natural antioxidants found in fruits and vegetables (*10*) that can fight the oxidative damage caused by free radicals, reducing the risk of chronic disease development (*11*).

In the meat industry, different chemical substances are commonly used, including nitrites, which increase shelf life and/or improve sensory characteristics (colour, taste and smell). Nitrites also play an important role in controlling the development of certain pathogenic microorganisms such as *Clostridium botulinum*, which is why they cannot be totally eliminated from the manufacture of meat products. Despite these benefits, nitrites have been rejected by some consumers since they are associated with substances that are harmful for humans. Nitrites react with secondary and tertiary amines to form N-nitroso derivatives, which can accelerate the development of certain terminal diseases (*12*). Therefore, alternatives of a natural origin are needed for the meat industry that can preserve products without altering the sensory characteristics or affecting the health of consumers.

The use of plant material as a source of functional compounds in the meat industry has been reported in numerous studies: the use of tomato pomace extract mixed with coriander essential oil (*13*); the use of celery powder, purple sweet potato powder, red gardenia powder, and paprika and blueberry (*14*); the application of powdered parsley extract in mortadella type sausage (*15*); and the addition of melon grain flour to beef sausages (*16*). However, there is no report on the use of bioactive compounds from guava epicarp and their application in meat products in order to reduce nitrite levels. Consequently, a new line of research related to the use and agro-industrial evaluation of guava residues and application of their bioactive compounds in the meat industry is needed. Therefore, the objective of the present study was to evaluate lipid extract from guava epicarp as an alternative to use of nitrites in the production of frankfurters and to evaluate their stability over time.

## MATERIALS AND METHODS

### Sausage preparation

This experiment was developed in the laboratories of Meat Technology and Fruit and Vegetable Technology of the National University of Colombia – Palmira. The raw materials were purchased in the regional market and a specialised meat supermarket in the city of Palmira, Valle del Cauca, Colombia. The guava residues (fruits at maturity degree 5) included epicarp, seeds and some mesocarp; fruits with visible mechanical damage or abnormal colouration were eliminated. The fruits were disinfected in a hypochlorite solution at 0.15 g/kg for 20 min and subsequently washed with potable water. Finally, an EGARVAC SCP Basic B (Vacarises, Barcelona, Spain) machine was used to pack the material in vacuum sealed polyethylene bags that were frozen at (-30±2) °C for 24 h.

The extract was prepared according to the ultrasound extraction method (*17*), in which the material was subjected to lyophilisation at vacuum pressure of 12 Pa and a condenser temperature of -80 °C for 24 h using an 18-litre tray (Labcomco, Kansas City MI, USA). The dry material was ground with an IKA M-20s3 mincer (Wilmington DE, USA) and sieved with a Ro-Tap Tyler RX-29-E (W.S. Tyler, Mentor, OH, USA) for 15 min until obtaining a flour with particles size of 0.074 mm. Then, to extract the carotenoids, sunflower oil was used as an extraction reagent, with the following operating conditions: ultrasound power 240 W, extraction time 40 min, process temperature 60 °C and flour/oil ratio 0.0256 g per 4 mL, in the Branson 1510 ultrasonic cleaner (Branson Ultrasonics Corp., Danbury CN, USA), obtaining a mass fraction of β-carotene of 47.40 mg/100 g extract. This ultrasound-assisted extraction allows a 36% higher yield than the conventional maceration.

The sausages were made with pork backfat. For the preparation of sausages, the formulation (Table 1) and the procedure were taken from Pinzón-Zárate *et al*. (*10*) and the Colombian Technical Standard NTC 1325 (*18*). Table 2 shows three treatments (three samples were taken for each treatment) with different mass fractions of guava epicarp flour extract and a control treatment.

**Table 1:** Frankfurter sausage formulation

Ingredient	*w*(ingredient)/%	
Pork meat (pH=6.3)	65	
Backfat	16	
Wheat flour	5	
Ice	10	
Additives	4	
Total	100	
Additive	*w*(additive)%	*m*(additive)/*m*(meat)/(g/kg)
Salt	0.846	7.0
Sugar	0.242	2.0
Garlic	0.459	3.8
Powdered onion	0.060	0.5
Pepper	0.060	0.5
Phosphates	0.484	4.0
Seasoning	1.208	10.0
Monosodium glutamate	0.121	1.0
Ascorbic acid	0.121	1.0
Nitrites	0.398	3.3
Total	4.000	33.1

**Table 2:** Mass fractions of nitrites and oily extract (carotenoids) of guava epicarp in processed sausages

Treatment	Sausage with nitrites		Sausage with oily extract			
*w*(nitrite)/%	*m*(nitrite)/*m*(meat)/(g/kg)		*w*(oily extract)/%		*m*(oily extract)/*m*(meat)/(g/kg)	
Control	100	3.300	0		0.000	
T_1_	75	2.475	25		0.825	
T_2_	50	1.650	50		1.650	
T_3_	25	0.825	75		2.475	

Lean pork meat (pH=6.3) and selected backfat, free of foreign odours, were used to make the sausages. Wheat flour was used as an extender and water was added in the form of ice to achieve the desired consistency of the final product. The additives included those traditionally accepted by the meat industry to improve the organoleptic characteristics and extend the shelf life of the final product. The maximum content of nitrites in the processed meat products was defined in accordance with the Colombian Technical Standard NTC 1325 (*18*).

Determination of physicochemical properties of sausages

Based on the AOAC methods (*19*), the dry matter, protein, fat, ash and carbohydrates were determined by difference from the proximate analysis, for each of the analysed treatments, and the caloric intake was determined according to the Berthelot-Malher bomb calorimeter method cited by Fabbri *et al*. (*20*) using Parr 6300 oxygen bomb calorimeter (Parr Instrument Company, Moline IL, USA). All analyses were done in triplicate.

The pH and water holding capacity (WHC) were determined according to the method proposed by Dzudie *et al*. (*21*). For the pH determination, a digital Metter Toledo MP 230 (Greifensee, Switzerland), was used and for the WHC, a sample of 0.5 g was taken from each sausage, placed on a grade 1 filter paper and subjected to pressure with a 1-kg plexiglass plate for 20 min. The surface area of the pressed sausage and the extracted liquid were defined using ImageJ (ImageJ^®^ v. 15 11) (*22*), and the WHC was calculated according to the following equations:


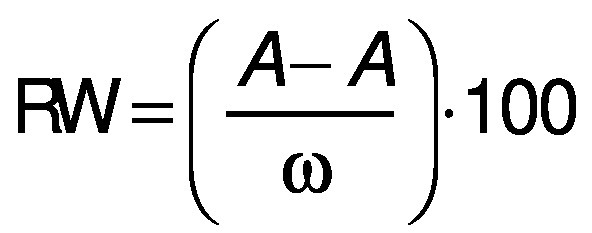



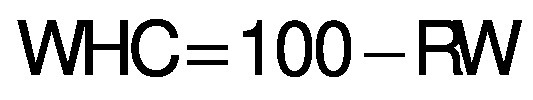


where RW is released water, *A*_1_ is the total surface area, *A*_2_ is the area of the pressed sausage, ω is the total moisture of the sausage sample and WHC is the water holding capacity.

The stability of the emulsion was determined according to Choe *et al*. (*23*). A mass of 6 g meat emulsion was placed in 16-mL Falcon tubes that were previously weighed, heated to (75±1) °C for 30 min and centrifuged at 2100×*g* for 5 min. A PCE-CFC 100 centrifuge (Instruments, Albacete, Spain) was used. The stability of the emulsion was calculated as follows:


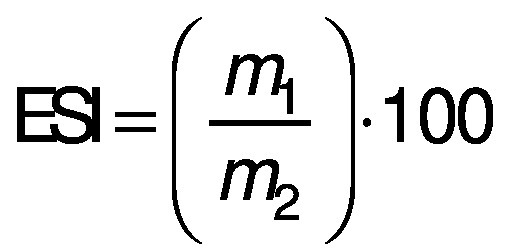


where ESI is the emulsion stability index (%), *m*_1_ is the mass (g) of the emulsion in the Falcon tube after drainage of the lipid layer and *m*_2_ is the mass (g) of the meat emulsion in the tube before heating.

The water activity of the different sausages was determined according to the AOAC method (*19*) with the help of an AQUALAB 4te hydrometer (METER Group, Inc., Pullman, WA, USA).

### Texture profile analysis

The Shimadzu EZ-SX food texture analyser (Tokyo, Japan) was used according to the method proposed by Savadkoohi *et al*. (*24*). A 15 mm thick slice of each of the analysed sausages was placed in the two parallel plates and compressed at 50% at a speed of 5 mm/min (three replications were made for each sample analysis). The hardness, cohesiveness, elasticity, chewiness, adhesiveness and gumminess of the samples were measured.

#### CIE L*a*b* colour coordinate determination

The colour coordinates were measured according to the method proposed by Ordóñez-Santos *et al*. (*25*). A Minolta Meter CR-100 (Tokyo, Japan) colourimeter was used. A D65 illuminant (8 mm diameter measurement area) and a 10° observer were used as reference (equipment calibrated with a white ceramic plate with reference values Y=89.5, x=0.3176 and y=0.3340). From each sausage, three cylindrical samples 5 cm in length were taken, from which a longitudinal incision was made to determine the internal colour. The luminosity (L*) and the coordinates (a* and b*) were measured, and based on these parameters the chromaticity or saturation index (C*), hue (*h* °) and total difference of colour (*∆E*) were determined, respectively, as follows:


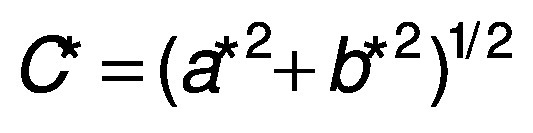



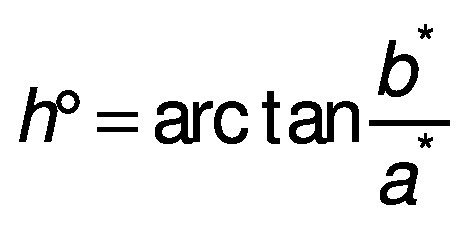



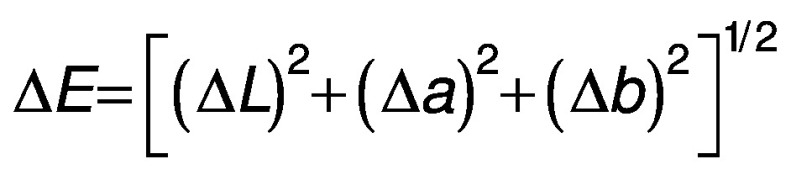


### Sensory evaluation of sausages

The sensory parameters smell, colour, flavour, texture and acceptability were evaluated by a panel consisting of 50 semi-trained evaluators of both genders (25 males and 25 females) and aged between 17 and 65. For the evaluation, the freshly prepared sausages were chopped into 1.5 cm long pieces and fried in sunflower vegetable oil. A seven-point unstructured hedonic scale where 1 means dislike very much and 7 means like very much was used (*26*).

### Stability analysis of sausages

The sausage treatment that showed the best results in terms of physicochemical, texture profile, colour and sensory parameters was taken, and its stability was analysed over time. The proximate analysis and colour coordinates were determined on days 0, 10, 20 and 30 of storage of vacuum-packed samples under refrigeration ((6±2) °C). Likewise, the residual nitrite content was analysed according to the method proposed by Zahran and Kassem (*27*), along with the oxidation of fats, which was based on the determination of 2-thiobarbituric acid reactive substances (TBARS), substances formed as a secondary product of lipid peroxidation (the values expressed as mass fraction of malondialdehyde (MDA)), the quantification of the *p*-anisidine value according to method proposed by Osawa *et al*. (*28*) and the peroxide index according to method proposed by Dermis *et al.* (*29*).

### Statistical analysis

This research was based on a simple randomised design, and the study of stability over time used a randomised block design. Data are presented as mean value±standard deviation of at least three replicate measurements. All statistical analyses and the effect of the variables and their interactions were evaluated with analysis of variance (ANOVA). The difference between the mean values of treatments was determined with the Tukey’s test with a probability for significant differences of p<0.05. The statistical analysis was performed using SPSS v. 22.0 (*30*).

## RESULTS AND DISCUSION

### Physicochemical properties of the sausages

Table 3 shows the results of the proximate analysis and the physicochemical properties of the three treatments. There were no significant differences (p<0.05), which led to the conclusion that the addition of the guava epicarp flour extract did not alter the chemical characteristics of the different sausages. Some studies have suggested the use of vegetable extracts to fully or partially replace nitrite and nitrate salts in sausages, without significantly affecting the characteristics of the product. The results were similar to those recorded by Riyad *et al*. (*31*), who added dry parsley, coriander and spinach powders to beef sausages. The physicochemical parameters pH, WHC, *a*_w_ and ESI of the different treatments compared with the control sausage did not show relevant differences. The pH values found in this research were lower than those obtained by Kim *et al*. (*32*) (pH=6.29 on average) in the sausage with konjac (*Amorphophallus konjac*) gel and powders from different vegetables. However, they were similar to those found in the study of Serdaroğlou and Őzsűmer (*33*), who measured pH=5.93 on average in sausages with soy protein, whey powder and wheat gluten. Chattopadhyay *et al*. (*34*) registered an average pH=6.55, higher than in the present study, in fish sausage with a vegetable gel, while the WHC value (91.41% on average) was lower than in the present study. Auremia *et al*. (*35*) worked with chicken mortadella type sausages with moringa seed flour and observed an average pH=5.92, slightly higher than in this study; however, the WHC values (96.23% on average), the stability of the emulsion (99.66% on average) and *a*_w_ (0.974) were similar to those obtained in this study. On the other hand, Savadkoohi *et al*. (*24*) worked with sausages with tomato bagasse and obtained values similar to those in this study (pH=5.48, WHC=88.22%). Finally, Rosero-Chasoy *et al*. (*36*) registered a pH=6.04 for sausages with yacon peel flour, higher than in the present study, and a value of 96.8% for the stability of the emulsion, similar to that obtained in the present study. The high variability in physicochemical parameters is attributed to the marked differences in the chemical composition of the different vegetables added to the meat products.

**Table 3:** Proximate analysis, physicochemical properties, texture profile and CIE L*a*b* colour coordinates of the processed sausages with different mass fractions of guava epicarp flour extract (guava extract)

Parameter	Control	*w*(guava extract in sausage)/%		
T_1_	T_2_	T_3_
0	25	50	75
Proximate analysis			
*w*(dry matter)/%	(31.6±0.3)^a^	(31.1±0.1)^a^	(32.1±1.2)^a^	(31.4± 0.3)^a^
*w*(protein)/%	(61.35±0.08)^a^	(60.6±0.2)^a^	(61.35±0.08)^a^	(61.35±0.08)^a^
*w*(fat)/%	(18.2±0.1)^a^	(18.2± 0.1)^a^	(18.2±0.1)^a^	(18.2±0.1)^a^
*w*(ash)/%	(7.8±0.3)^a^	(8.3±0.3)^a^	(7.8±0.4)^a^	(7.8± 0.5)^a^
*E*/(kJ/g)	(22.7±0.6)^a^	(22.2±0.2)^a^	(22.7±0.6)^a^	(22.7±0.1)^a^
	Physicochemical property			
pH	(5.4±0.3)^a^	(5.5±1.1)^a^	(5.4±0.8)^a^	(5.4±0.8)^a^
WHC/%	(95.8±0.6)^a^	(95.6±0.1)^a^	(96.0±0.4)^a^	(96.1±0.2)^a^
*a*_w_	(1.0±1.4)^a^	(1.0±0.3)^a^	(1.0±0.6)^a^	(1.0±0.4)^a^
ESI/%	(98.0±0.3)^a^	(99.2±1.0)^a^	(98.8±0.3)^a^	(98.9±1.0)^a^
	Texture profile			
Hardness/N	(41.6±0.2)^a^	(41.0± 0.2)^a^	(41.8±0.8)^a^	(40.6±0.1)^a^
Cohesiveness	(-0.01±0.73)^a^	(-0.01±0.91)^a^	(-0.01±0.86)^a^	(-0.01±0.26)^a^
Elasticity/mm	(1.0±1.2)^a^	(0.8±1.9)^b^	(0.9±0.5)^b^	(0.9±0.6)^a^
Chewiness/(N/mm)	(22.3±0.1)^a^	(23.1±0.08)^a^	(22.9±0.4)^a^	(22.4±0.2)^a^
Adhesiveness/(N/mm)	(-0.4±0.6)^a^	(-0.3±0.1)^a^	(-0.3±0.2)^a^	(-0.3±0.5)^a^
Gumminess/N	(9.6±0.3)^a^	(9.9±0.4)^a^	(10.2±0.6)^b^	(10.0±0.6)^b^
	CIE L*a*b* colour coordinate			
L*	(75.7±0.6)^a^	(75.4±0.4)^a^	(77.4±0.4)^b^	(77.7±0.5)^b^
a*	(3.8±0.2)^a^	(4.5±0.2)^b^	(4.09± }0.07)^b^	(3.1±0.2)^a^
b*	(10.01±0.2)^a^	(10.3±0.09)^a^	(10.7±0.3)^a^	(10.7±0.3)^a^
C*	(10.8±0.2)^a^	(11.25±0.03)^b^	(11.4±0.2)^b^	(11.2±0.3)^b^
*h*°	(69.0±1.2)^a^	(66.38±1.09)^b^	(69.0±0.8)^a^	(73.9±0.6)^c^
Δ*E*	-	(0.8±0.2)	(1.9±0.4)	(2.3±0.4)

WCH=water holding capacity, ESI=emulsion stability index

L*: 0=black and 100=white, a*: -60=green and +60=red, b*: -60=blue and +60=yellow, *h*=tone angle: 90°=yellow, 180°=green and 0°=red, C*=saturation index, distance from the coordinates at the origin to the determined colour point, ∆*E*=total difference of colour

The results are shown as mean value±S.D. (*N*=3)

Different letters in superscript in the same row indicate that there is a significant effect between the samples, p<0.05

### Determination of the texture profile and CIE L*a*b* colour coordinates

Table 3 also shows the results of colour and texture determinations. The elasticity and gumminess values showed statistical differences, while for the other parameters, no impact was observed on the texture of the sausages. The different size of the fat particles, especially those with a larger diameter, led to a reduction in the adhesion between proteins and cellulose and the proteins and fat globules in the meat emulsion, which influenced the texture of the sausages (*37*).

When comparing the values with those found by Saldaña *et al*. (*38*), who worked with low-fat mortadella type sausages, lower values were found in the present study for hardness (16.24 N), elasticity (0.86 mm) and chewiness (11.49 N/mm). In another research developed by Ozaki *et al*. (39) using sausages with two mass fractions of a mixture of radish powder and chitosan, higher values were found than in this study, except for adhesiveness. Finally, the results in sausages with tomato bagasse obtained by Savadkoohi *et al*. (*24*) were lower for hardness (27.95 N), while for elasticity (3.49 mm), adhesiveness (0.22 N/mm), cohesiveness (0.41 N) and chewiness (40.05 N/mm) higher values were found than in this study. The differences in the texture parameters shown in the different studies are probably attributed to the variety in the composition of the sausages and particularly to the characteristics and quantities of fats, water and hydrocolloids used in their preparation.

For the colour coordinates, no significant differences were detected in the b* parameter; the other parameters had significant differences in relation to the control sausage. For the CIE L*a*b* parameters, the colouration depends on a high degree of the luminosity (L*) and the red-green coordinate (a*). In the present study, the luminosity was directly proportional to the mass fraction of the guava extract added to the sausages, while coordinate a* had slight downward trend, which is explained by the characteristics of the added carotenoid pigments. These results differ from those noted by Syuhairah *et al*. (*40*) for colour parameters in chicken sausages with different mass fractions of spinach, purple cabbage, carrot, pepper and mushroom: L* and b* coordinates had higher values, while coordinate a* varied depending on the mass fraction applied, which is explained by the presence of more or less natural pigments in the formulations used to prepare the sausages. De Carvalho *et al*. (*41*) combined lamb sausages with different vegetable oils, and obtained values of L*=69.01, a*=8.52 and b*=17.38, where the luminosity was lower but coordinates a* and b* were higher than in the present study. The high variability in the luminosity parameter (L*) is attributed to the fact that the concentration of the pigments in the different formulations of the sausages does not have a direct impact on the luminosity but rather this parameter is basically influenced by the content of bound water and the amount of fat present in the empty intercellular spaces, which leads to a decrease in the oxidation capacity of myoglobin. Variations in the a* coordinate in the different studies may be due to the formation of a larger or smaller total amount of nitrosomyoglobin and the red pigments deoxymyoglobin and oxymyoglobin in the sausages, and consequently the addition of the guava extract, which contributes certain pigments that do not promote the development of a high red-green colouration.

For saturation index (C*), a significant increase was not observed among the three treatments, while compared to the control treatment, there was an increase of 4.35, 5.93 and 3.71% respectively. On the contrary, the hue angle (*h*°) increased as the nitrite content decrease and the guava extract mass fraction increased, which can be explained by the reduction of nitrite to nitric oxide and the subsequent reaction with myoglobin, forming nitrosomyoglobin, which stabilises during heat treatments. The values obtained in this research coincide with those shown by Pinzón-Zárate *et al*. (*10*) in sausages with chontaduro palm (*Bactris gasipaes*) extract and by Rosero-Chasoy *et al*. (*36*) in sausages with yacon peel flour.

Finally, the differences in colour (∆*E*) were minimal compared to the control sausage, without exceeding the value of 0.3, reported as the lowest accepted value that shows significant differences, the lowest value found in the sausage with 25% nitrites replaced by guava epicarp (0.83 in T_1_).

### Sensory properties of the sausages

**Fig. 1:**
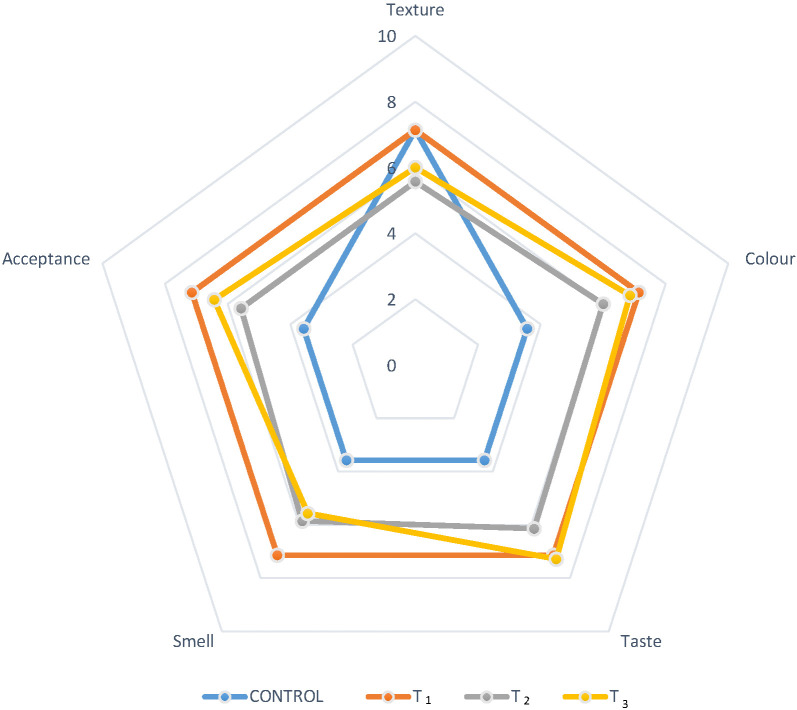
Sensory attributes of sausages with guava epicarp flour extract (guava extract). *w*(guava extract)/%: control=0, T_1_= 25, T_2_= 50, T_3_= 75. Quality scale: 7=like very much, 6=like, 5=like moderately, 4=neither like nor dislike, 3=dislike moderately, 2=dislike,1=dislike very much

Fig. 1 shows the results of the sensory analysis. According to the panellists, texture, taste, smell and acceptance improved with the addition of the three mass fractions of guava extract, while the score for texture remained similar to that of the control sausage. However, the formulation with the highest acceptability was T_1_, while higher mass fraction of guava extract led to a decrease in the acceptance by the panellists.

### Stability analysis of the selected sausages over time

The analysis showed that the sausages from T_1_ treatment (nitrite replaced by 25% guava extract) had better attributes and better acceptance according to their physicochemical, sensory, texture and colour properties. That is why they were used for the stability analysis during storage.

The proximate analysis did not reveal statistically significant differences comparing the results of any of the analysed components on day 0 with day 30 (Table 4).

**Table 4:** Proximate analysis and CIE L*a*b* colour parameters for the sausages with 25% guava epicarp flour extract and 75% nitrites during storage

Parameter	*t*/day			
0	10	20	30
Proximate analysis			
*w*(dry matter)/%	(31.1±0.1)^a^	(31.2±0.3)^a^	(31.2±0.3)^a^	(31.1±0.2)^a^
*w*(protein)/%	(60.6±0.1)^a^	(60.6±0.4)^a^	(60.6±0.3)^a^	(60.5±0.3)^a^
*w*(fat)/%	(18.2±0.2)^a^	(18.1±0.2)^a^	(18.1±0.3)^a^	(18.0±0.2)^a^
*w*(ash)/%	(8.3±0.3)^a^	(8.3±0.1)^a^	(8.3±0.2)^a^	(8.3±0.3)^a^
*E*/(kJ/g)	(22.2±0.2)^a^	(22.2±0.3)^a^	(22.2±0.3)^a^	(22.1±0.4)^a^
	CIE L*a*b* colour parameter			
L*	(72.5±0.0)^a^	(72.1±0.3)^a^	(72.1±0.6)^a^	(72.5±0.2)^a^
a*	(9.78±0.01)^a^	(9.41±0.07)^a^	(9.8±0.2)^a^	(9.76±0.03)^a^
b*	(8.36±0.07)^a^	(8.7±0.2)^a^	(8.7±0.3)^a^	(9.84±0.09)^b^
C*	(11.86±0.05)^a^	(12.8±0.2)^a^	(13.1±0.5)^b^	(13.86±0.30)^b^
*h*°	(40.1±0.2)^a^	(42.8±0.4)^b^	(41.5±0.5)^c^	(45.22±0.30)^c^
Δ*E*	-	(0.7±0.1)^a^	(0.61±0.03)^a^	(1.6±0.1)^b^

L*: 0=black and 100=white, a*: -60=green and +60=red, b*: -60=blue and +60=yellow, *h*°=tone angle: 90°=yellow, 180°=green and 0°=red, C*=saturation index, distance from the coordinates at the origin to the determined colour point. The results are shown as mean±S.D. (*N*=3)

Different letters in superscript in the same row indicate a significant difference between the samples, p<0.05

The CIE L*a*b* parameters did not show significant differences (p<0.05) for L* and a* coordinates, while b* coordinate, saturation index C*, hue angle *h*° and colour difference in relation to the control sausage Δ*E* showed differences as the storage time increased (Table 4). Colour is an important sensory parameter in the acceptance of food products but it also shows alterations that the product has undergone during storage. Table 4 shows a direct relationship between the storage time and the colour difference, which is attributed to the isomerisation of the carotenoid pigments present in the sausages. Several studies have presented results similar to those shown in this research: Ozaki *et al*. (*39*), who worked on sausages with pork and beef with a mixture of chitosan and radish powder for 60 days of storage, Souissi *et al*. (*42*), who worked with sausages made with octopus (*Octopus vulgaris*) meat and cuttlefish gel stored for 15 days, and Jin *et al*. (*14*), who worked with sausages with different vegetables as partial replacement for nitrites during four weeks of storage. This increase in the colour difference can be attributed to the bioactive compounds present in the added plant extracts, which promote the reaction between nitrite and myoglobin in muscle, leading to the formation of a red nitrosylhemochrome pigment (*43*).

According to Colombian legislation, residual nitrites in processed meat products should not exceed 150 mg/kg (*18*), which was met in the sausages analysed in this research. The analysis of this parameter showed an important reduction during storage (Table 5) as the result of the interaction between muscle myoglobin and nitrite transformed to NO, which results from oxidation reactions and the consumption of nitrite, causing the formation of NO and nitrosomyoglobin, *i.e.* the pink colour of sausages. The results agree with those of Jin *et al*. (*14*) for sausages with different vegetables at different mass fractions, obtaining a decrease from 27.90 to 17.22 mg/kg during four weeks of storage, and with those of Šojić *et al*. (*13*) who achieved a decrease of 24.60 mg/kg in pork sausage with coriander essential oil during 15 weeks of storage.

**Table 5:** Results of the analysis of residual nitrite and lipid oxidation in the sausages with 25% guava epicarp flour extract and 75% nitrite during storage

Parameter		*t*/day		
0	15	30
*w*(residual nitrite)/(mg/kg)		(42.2±0.4)^a^	(31.3±0.2)^b^	(23.6±0.2)^c^
Peroxide index/(mmol/kg)		(8.2±0.2)^a^	(11.6±0.8)^b^	(13.5±0.8)^b^
*p*-anisidine value/(µmol/µg)		(20.1±0.3)^a^	(23.9±0.4)^b^	(26.0±0.1)^b^
*w*(total volatile bases as N_2_)/(mg /100 g)		(14.9±0.7)^a^	(15.2±0.4)^b^	(16.1±0.4)^b^
TBARS (as MDA)/(mg/kg)		(0.41±0.04)^a^	(0.43±0.09)^b^	(0.46±0.07)^b^

TBARS=thiobarbituric acid reactive substances, MDA=malondialdehyde

The results are shown as mean value±S.D. (*N*=3)

Different letters in superscript in the same row indicate that there is a significant difference between the samples, p<0.05

During storage, changes may occur that lead to deterioration and/or rejection of the final product. These changes are mainly related to the oxidation of intramuscular fats, changes that affect the primary and secondary metabolites of fat oxidation. The data in Table 5 showed that, as the storage time increased, all measured parameters increased; however, statistically significant growth (p<0.05) was noted up to the first 15 days of storage, which led to the conclusion that the oxidation of fats can be decisive during this period.

## CONCLUSIONS

In this work, sausages were prepared with the addition of guava epicarp flour extract as a partial replacement for nitrite. The processed sausages showed very good physicochemical and sensory parameters, in addition, to the fact there were no alterations in the colour of the final products. The stability test showed no alterations in the quality of the samples up to 30 days. Therefore, it can be concluded that guava by-product, basically the epicarp, is an important alternative to the use of nitrites in the meat industry. Replacing up to 25% of nitrites in frankfurters with this extract, which is basically made up of natural carotenoid pigments, can help minimise the negative effects of nitrites on the health and well-being of consumers.
